# Limited effects of plant-beneficial fungi on plant volatile composition and host-choice behavior of *Nesidiocoris tenuis*

**DOI:** 10.3389/fpls.2023.1322719

**Published:** 2024-01-03

**Authors:** Caroline Meesters, Berhane T. Weldegergis, Marcel Dicke, Hans Jacquemyn, Bart Lievens

**Affiliations:** ^1^ Laboratory for Process Microbial Ecology and Bioinspirational Management (PME&BIM), Department of Microbial and Molecular Systems (M^2^S), KU Leuven, Leuven, Belgium; ^2^ Leuven Plant Institute (LPI), KU Leuven, Leuven, Belgium; ^3^ Laboratory of Entomology, Wageningen University & Research, Wageningen, Netherlands; ^4^ Laboratory of Plant Conservation and Population Biology, Biology Department, KU Leuven, Leuven, Belgium

**Keywords:** *Beauveria bassiana*, biological control, host-searching behavior, *Metarhizium brunneum*, *Trichoderma harzianum*

## Abstract

Biological control using plant-beneficial fungi has gained considerable interest as a sustainable method for pest management, by priming the plant for enhanced defense against pathogens and insect herbivores. However, despite promising outcomes, little is known about how different fungal strains mediate these beneficial effects. In this study, we evaluated whether inoculation of tomato seeds with the plant-beneficial fungi *Beauveria bassiana* ARSEF 3097, *Metarhizium brunneum* ARSEF 1095 and *Trichoderma harzianum* T22 affected the plant’s volatile organic compound (VOC) profile and the host-choice behavior of *Nesidiocoris tenuis*, an emerging pest species in NW-European tomato cultivation, and the related zoophytophagous biocontrol agent *Macrolophus pygmaeus*. Results indicated that fungal inoculation did not significantly alter the VOC composition of tomato plants. However, in a two-choice cage assay where female insects were given the option to select between control plants and fungus-inoculated plants, *N. tenuis* preferred control plants over *M. brunneum*-inoculated plants. Nearly 72% of all *N. tenuis* individuals tested chose the control treatment. In all other combinations tested, no significant differences were found for none of the insects. We conclude that inoculation of tomato with plant-beneficial fungi had limited effects on plant volatile composition and host-choice behavior of insects. However, the observation that *N. tenuis* was deterred from the crop when inoculated with *M. brunneum* and attracted to non-inoculated plants may provide new opportunities for future biocontrol based on a push-pull strategy.

## 1 Introduction

Plant diseases and pests pose significant threats to agricultural productivity and food security (Savary et al., 2019). Arthropods are estimated to destroy 18 – 26% of global annual crop production, equal to a value of more than 430 billion euro (Culliney, 2014). Classically, pests are controlled by the use of chemical pesticides, which have greatly benefited global agriculture and food security, but also posed risks to environmental sustainability and public health (Damalas and Eleftherohorinos, 2011; Sharma et al., 2019; Calvo-Agudo et al., 2021). In addition, the intensive use of chemical pesticides has resulted in the development of resistant pests and pathogens (Bass et al., 2015; Gould et al., 2018; Hawkins et al., 2019). To mitigate these negative impacts, substantial efforts have been made to reduce the use of chemical pesticides (cfr. EU-directives such as 91/414/EEC and the Farm to Fork strategy committed to work towards reducing the overall use and risk of chemical pesticides by 50% by 2030 (Parisi et al., 2015; European Commission, 2020; Silva et al., 2022)), and encourage more eco-friendly pest management methods like Integrated Pest Management (IPM) and biological control.

Among various options, biological control using plant-beneficial microbes has gained considerable importance as a method for pest management, by priming the plant for enhanced defense against pathogens and insect herbivores (Pieterse et al., 2014). Beneficial microbes in the root microbiome contribute positively to plant growth and performance through direct and indirect mechanisms (Van der Putten et al., 2001; Wardle et al., 2004; Bezemer and Van Dam, 2005). Direct effects may result from improved availability and uptake of nutrients (Meesters et al., 2023), and by exerting direct negative effects on the behavior or performance of herbivorous insects, e.g. by the production of repellants, antifeedants and toxins (Pineda et al., 2010). Indirect effects may result from enhanced recruitment of natural enemies of herbivores or improving their activity (Pineda et al., 2010). Plant inoculation with beneficial microbes can alter the physiology of the plant, leading to changes in the plant-volatile profile (Shikano et al., 2017; Vega, 2018), which in turn can affect higher trophic levels such as insect herbivores and their natural enemies. For example, inoculation of tomato plants with the endophytic fungus *Fusarium solani* Saccardo strain K (Hypocreales: Nectriaceae) increased both direct and indirect tomato defenses against spider mites, by directly reducing their performance and attracting more predators, respectively, when compared with control plants (Garantonakis et al., 2018). Similarly, endophytic colonization by the entomopathogenic fungus *Beauveria bassiana* Vuillemin strain EABb 01/33-Su (Hypocreales: Cordycipitaceae) altered the composition of the volatile organic compounds (VOCs) emitted by melon and cotton plants. Some of the emitted compounds are known to be released in response to herbivore attack and have been implicated in natural enemy attraction (González-Mas et al., 2021). Likewise, tomato inoculation with *Trichoderma longibrachiatum* Rifai strain MK1 (Hypocreales: Hypocreaceae) increased the plant’s attractiveness towards the generalist predator *Macrolophus pygmaeus* Rambur (Hemiptera: Miridae) (Battaglia et al., 2013). However, effects of plant inoculation with beneficial microbes on herbivorous insects and their natural enemies have been found to be ambiguous: in some cases, herbivores are repelled by microbe-inoculated plants (Rondot and Reineke, 2017; Sword et al., 2017; Wei et al., 2020), while in other cases they are attracted (Wilberts et al., 2022). Similarly, natural enemies are attracted in some cases (Battaglia et al., 2013; Pangesti et al., 2015) while repelled in others (Ormond et al., 2011). So far, only little is known about the factors determining microbe-mediated plant responses, but it is generally assumed that fungal species or strain, along with plant species or genotype (cultivar), and insect species, play an important role in this context (Berg, 2009; Alam et al., 2021). Nevertheless, most studies performed so far have focused on a single fungal strain (Battaglia et al., 2013; Garantonakis et al., 2018; Alınç et al., 2021), making it difficult to ascertain how the interactions between plants and insects are mediated by different fungal strains.

The main goal of this study was to investigate the effects of different plant-beneficial fungi on plant volatile emissions and how this affects the host-choice behavior of plant-feeding insects. Specifically, we assessed the effects of seed inoculation of tomato *Solanum lycopersicum* Linnaeus (Solanales: Solanaceae) with the fungal strains *Beauveria bassiana* ARSEF 3097, *Metarhizium brunneum* ARSEF 1095 Petch (Hypocreales: Cordycipitaceae) and *Trichoderma harzianum* Rifai T22 (Hypocreales: Hypocreaceae) on the plant VOC profile and the host-choice behavior of *Nesidiocoris tenuis* Reuter (Hemiptera: Miridae) and the related zoophytophagous mirid bug *M. pygmaeus*. While *N. tenuis* is an important biological control agent of whiteflies, leafminers, thrips and spider mites in Mediterranean countries (Sanchez, 2008), in recent years it has become an important problem in the greenhouse cultivation of tomatoes in Northwestern Europe, especially when *N. tenuis* population densities are high or when prey is scarce or absent (Siscaro et al., 2019). Plant feeding by *N. tenuis* causes the formation of necrotic rings on stems and leaf petioles, resulting in flower abortion in addition to punctures in fruits. These effects subsequently result in reduced crop quality and yield (Siscaro et al., 2019). By contrast, the phylogenetically related mirid *M. pygmaeus* is less harmful and is commonly used as a biocontrol agent against various herbivorous insects like the tomato borer *Tuta absoluta* Meyrick (Lepidoptera: Gelechiidae), whiteflies, thrips, leaf miners, aphids, spider mites and lepidopterans (Calvo et al., 2009; Nannini et al., 2012; Perdikis et al., 2014). Ultimately, we aimed to identify fungal strains that deter *N. tenuis*, while having no effect on *M. pygmaeus*. Such strains could provide a promising approach to enhance the biocontrol of *N. tenuis*.

## 2 Materials and methods

### 2.1 Study organisms

*Beauveria bassiana* ARSEF 3097 and *Metarhizium brunneum* ARSEF 1095 are the active ingredients of the commercially available bioinsecticides Naturalis^®^ and BIPESCO^®^, respectively. These strains were obtained from the Agricultural Research Service Collection of Entomopathogenic Fungal Cultures (ARSEF) located in New York, USA. *Trichoderma harzianum* strain T22, which has recently been re-classified as *Trichoderma afroharzianum* Rifai (Hypocreales: Hypocreaceae) (Chaverri et al., 2015) (for consistency with previous research further referred to as *T. harzianum* in this study) is the active ingredient in various biofertilizers and biopesticides, including Trianum-P (Koppert Biological Systems, The Netherlands), from which it was isolated for this study. While predominantly known as entomopathogenic fungi, *B. bassiana* ARSEF 3097 and *M. brunneum* ARSEF 1095 are also able to endophytically colonize plant tissues from several hosts upon artificial inoculation, including tomato (Klieber and Reineke, 2016; Jaber and Araj, 2018; Wilberts et al., 2022; Meesters et al., 2023). *Trichoderma harzianum* T22 is primarily known to colonize plant roots epiphytically but has also been found to colonize plant tissues endophytically (Harman et al., 2004). The three selected strains have been found to offer plants various benefits, including enhanced plant growth (Sani et al., 2020; Van Hee et al., 2023; Wilberts et al., 2023) and increased resistance against pathogens and/or herbivorous insects (Jaber and Alananbeh, 2018; Jaber and Araj, 2018; Alınç et al., 2021; Wilberts et al., 2022; Meesters et al., 2023; Van Hee et al., 2023). The fungal strains were preserved on potato dextrose agar (PDA) plugs in 35% glycerol at -80°C until further use. Both a lab culture of *N. tenuis* and *M. pygmaeus* were established using adult specimens kindly provided by Biobest N.V. (Westerlo, Belgium). Lab cultures were then maintained in mesh insect cages (17.5 cm × 17.5 cm × 17.5 cm, 96 × 26 mesh - 680 µm aperture, BugDorm, MegaView Science Co., Ltd.) under controlled conditions (25 ± 1°C, 70 ± 10% relative humidity (RH) and 16L:8D photoperiod; ECL02, Snijders Labs, The Netherlands)). Insects were provided with *ad libitum* access to γ-irradiated *Ephestia kuehniella* Zeller (Lepidoptera: Pyralidae) eggs as a food source without plants. Wet cotton sticks enclosed in stretched Parafilm^®^ were offered as oviposition substrate and water source (De Puysseleyr et al., 2013).

### 2.2 Fungal inoculation

Plants were inoculated as previously described (Soad et al., 2005; Meesters et al., 2023). Initially, stock cultures of the three fungi were plated on agar-based quarter-strength (¼) Sabouraud dextrose agar, which was supplemented with yeast extract (SDAY) and subsequently transferred to the same medium again. Next, the fungal strains were grown on SDAY at a temperature of 25°C. Following a ten-day incubation period, conidia were collected by gently scraping the spores from the agar plates after being flooded with sterile physiological saline solution (0.8% w/v NaCl). The obtained suspension was filtered through a sterile microcloth (Mira Cloth, Merck, Massachusetts, USA) and washed twice using sterile physiological saline solution to yield a purified suspension of fungal conidia. Once the number of conidia was counted using a Bürker haemocytometer, the spore concentration was adjusted to 1 × 10^7^ spores mL^-1^. Before conducting experiments, conidial viability was assessed by plating a 100 µL aliquot of 1 × 10^3^ conidia mL^-1^ on three SDAY plates. Following incubation at 25°C for 24 hours, the numbers of germinated and ungerminated conidia were counted under a microscope. Spores were considered germinated when the germ tube extended to a length at least twice that of the spore diameter. Results of the germination tests demonstrated > 90% viability rate for all conidial suspensions.

Prior to inoculation, tomato seeds were surface-sterilized using a 1% (v/v) sodium hypochlorite solution for 5 min with agitation, followed by rinsing four times using sterile distilled water. Subsequently, 25 surface-sterilized seeds were put on a filter paper inside a 9 cm diameter Petri dish and then moistened with 2 mL sterile distilled water. Plates were wrapped with breathable surgical tape (3M, Belgium) and incubated in the dark at 25°C for 48 h to promote germination. Germinated seeds were submerged for 24 h by addition of 6 mL conidial suspension into the Petri dishes (Soad et al., 2005) or using physiological water as a control. Germinated seeds were then planted in a growth medium comprising a 3:1 ratio of potting mix (Universal potting mix; Agrofino, Ghent, Belgium) and white sand (for chemical characteristics of the potting mixture, see **Supplementary Table 1**, Supporting Information), after which they were put in a plant growth cabinet (MD1400, Snijders Labs, The Netherlands) at 23 ± 1°C, 65 ± 2% RH and a 16L:8D photoperiod. The growth cabinet was equipped with white LED lights that provided a photosynthetic flux density of 220 µmol photons m^-2^ s^-1^. After 14 days, seedlings were individually transplanted in 10.5 cm diameter plastic pots with the same potting mix as mentioned earlier and placed in a climate-controlled greenhouse compartment until further use. Plants were put together according to fungal treatment to avoid contamination between the different treatments at a distance of at least 50 cm between treatments. When transplanting the plants, fungal colonization was also verified by collecting root samples from three plants per treatment (not further used in the experiments), as described in Meesters et al. (2023), and showed that the inoculation was successful.

### 2.3 Collection and analysis of plant volatile organic compounds

Seven weeks after fungal inoculation, tomato plants were subjected to dynamic headspace sampling to assess the VOC composition of their aboveground plant parts. At that time, no visual differences were observed among fungus-treated and control plants. For each treatment, individual tomato plants (*n* = 10) were enclosed within a glass dome (height: 20 cm; diameter: 23 cm), which was sealed with aluminum plates around the stem right above the first true leaf, while ensuring that the plant was not constricted. Volatiles collected from empty glass domes were used as background volatiles. Glass domes were cleaned with acetone and heated at 175°C for 2 h before being used for plant volatile collections. To maintain a positive pressure within the domes, charcoal-filtered air was pumped into each dome at 250 mL min^-1^ and drawn out at 200 mL min^-1^ through a stainless-steel tube filled with 200 mg Tenax TA adsorbent (20/35 mesh; CAMSCO, Houston, TX, USA). Collections were carried out under laboratory conditions (23  ±  2°C; 65  ±  5% RH; 16L:8D photoperiod) for a period of 2 h (lights on), after acclimatization of the plants to the room for 24 hours and for 30 min in the glass domes. Desorption of volatiles from the Tenax TA adsorbent as well as subsequent separation and detection of the volatiles were conducted using a Thermal Desorber TD100-xr (Markes, Llantrisant, Glamorgan, UK), which was connected to a 7890B gas chromatograph (GC) coupled to quadrupole-time-of-flight mass spectrometer (Q-ToF) (both from Agilent (Agilent Technologies, USA)). Volatiles were released from the adsorbent at 250°C for 10 min under a helium flow rate of 30 mL min^-1^ and simultaneously re-collected in an electronically cooled solvent trap (Markes) at 0°C. Following completion of the desorption and re-collection process, volatiles were released from the cold trap by ballistic heating at a rate of 40°C s^-1^ to 280°C, which was then kept for 5 min, while the volatiles were directed to a DB-5MS analytical column (Phenomenex, Torrance, CA, USA) (length: 30 m; inner diameter: 0.25 mm; film thickness: 1 µm), placed inside the oven of the GC at a split ratio of 100:1 for further separation. Initially, the GC oven temperature was held at 40°C for 2 min, then raised at a rate of 10°C min^-1^ to 100°C, and then held for 1 min. Then it was raised at a rate of 5°C min^-1^ to 140°C and immediately thereafter at a rate of 10°C min^-1^ to a final temperature of 280°C, where it was kept for 1 min under a constant helium flow of 1.2 mL min^-1^. Column effluents were ionized through electron impact ionization at 70 eV and subsequently detected with an accurate mass Q-ToF MS, acquiring mass spectra from 35- 400 m/z at an acquisition rate of 5 spectra s^-1^. The transfer line and ion source of the Q-ToF MS were set at 280°C and 230°C, respectively. To detect the presence of plant volatile compounds, chromatograms were recorded using MassHunter deconvolution software (Agilent Techologies, Inc 2008). Next, chromatograms were converted to Xcalibur data through a two-step raw data conversion program provided within the MetAlign software (Lommen, 2009). Automated baseline correction, peak selection (Signal-to-Noise ratio > 3) and alignments of all extracted mass signals of the raw data were processed following an untargeted metabolomic workflow using MetAlign, which provides detailed information on the abundance of the mass signals representing the various volatile compounds (Lommen, 2009). Next, the extracted mass features were reconstructed into potential compounds using the MSClust software through data reduction employing unsupervised clustering and extraction of putative metabolite mass spectra (Tikunov et al., 2012). Tentative identification of volatile compounds relied on a comparative analysis of the reconstructed mass spectra with those in the NIST 2014 and Wageningen Mass Spectral Database of Natural Products MS libraries. In addition, experimentally obtained linear retention indices (LRIs) were used as an additional element in the identification process.

### 2.4 Two-choice cage assay

Immediately after VOC collection, plants were subjected to a two-choice cage bioassay, set up according to Battaglia et al. (2013), to assess plant attractiveness to *N. tenuis* and *M. pygmaeus* and evaluate their host preference. Experiments were performed in nylon mesh insect cages (60 cm × 40 cm × 40 cm (W × L × H), mesh size 0.25 mm × 0.25 mm, Entomologie-Speciaalzaak Vermandel V.O.F., The Netherlands). Both an inoculated and non-inoculated control plant were put in each cage at a distance of 30 cm from each other (avoiding any contact between the two plants). Ten *N. tenuis* or *M. pygmaeus* adult females that were less than one week in the adult stage were introduced in the middle of each cage at an equal distance from both plants. Insects were not provided with additional food or prey in order to stimulate host selection and plant feeding. Prior to subjecting the insects to the experiments, insects were starved for one day by providing only water and putting each of them in individual transparent plastic containers (1.5 cm × 1.5 cm × 1.5 cm) to prevent cannibalism. Twenty-four hours after their release, insect positions were recorded by visual inspection of each plant. As a control, insects were also given the choice between two non-inoculated control plants. The experiment was performed at five time points, with two replicates per time point (*n* = 10, except for plants inoculated with *B. bassiana* ARSEF 3097 exposed to *M. pygmaeus*; *n* = 9). Cages were set-up in a glass greenhouse compartment with climate control (20 ± 4°C, RH = 66 ± 20%, and a 18L:6D photoperiod; **Supplementary Figure 1**), over a time period of 10 days in March 2022 using a fully randomized block design to avoid spatial effects.

### 2.5 Statistical analysis

A Principal Component Analysis (PCA) using the VOC peak heights correlation matrix was performed to visualize differences in the plant VOC composition between fungal strains. To assess whether the chemical composition of the VOC blends differed significantly among fungal strains, a one-way permutational multivariate analysis of variance (perMANOVA) was performed on the data matrix with fungal strain and VOC peak heights. The assessment of statistical significance was based on 1000 permutations. The analysis was executed using the adonis2 function of the vegan package in R. To further assess differences in the VOC composition between the different treatments at the level of compounds, a univariate ANOVA or Kruskal Wallis test (when the normality assumption was not met) was performed on the VOC peak heights of the different compounds. Likewise, to assess differences between the control treatment and each of the fungal treatments, pair-wise *post-hoc* tests were performed on the VOC peak heights using the Student’s *t*-test or Wilcoxon Rank Sum test. All these statistical analyses were performed in R 4.0.3 (R Core Team, 2013).

To analyze insect response, for each tested combination, we employed a generalized linear mixed model (GLMM) with a binomial distribution (choice is binary: for either control side or treatment side) with a logit link function (logistic regression). Fungal strain was used as a fixed factor, utilizing the ‘glmer’ function from the lme4 package in R. In this analysis, each release of one cohort of ten insects (*n* = 10; except for *B. bassiana*-inoculated plants exposed to *M. pygmaeus* for which *n* = 9) was considered as a replicate. To prevent overdispersion and mitigate pseudoreplication, we incorporated the release of each cohort as a random factor in the model, as well as the day of the experiment. The response variable in the model was the number of insects choosing the control or treatment side of each cohort. Subsequently, we performed an analysis of variance type III χ2-test on the GLMM to determine whether there was an overall difference between the responses to the different tested fungal strains. Next, pair-wise *post hoc* tests (with estimated marginal means performed with the ‘Emmeans’ package) were used to determine differences between control and fungal treatments. Results were presented by calculating the percentage of insects choosing fungus-inoculated or non-inoculated (control) plants. Insects located on other places than the plant were considered non-responders and not taken into account in the statistical analyses.

## 3 Results

### 3.1 Effect on plant VOC composition

**Figure 1:**
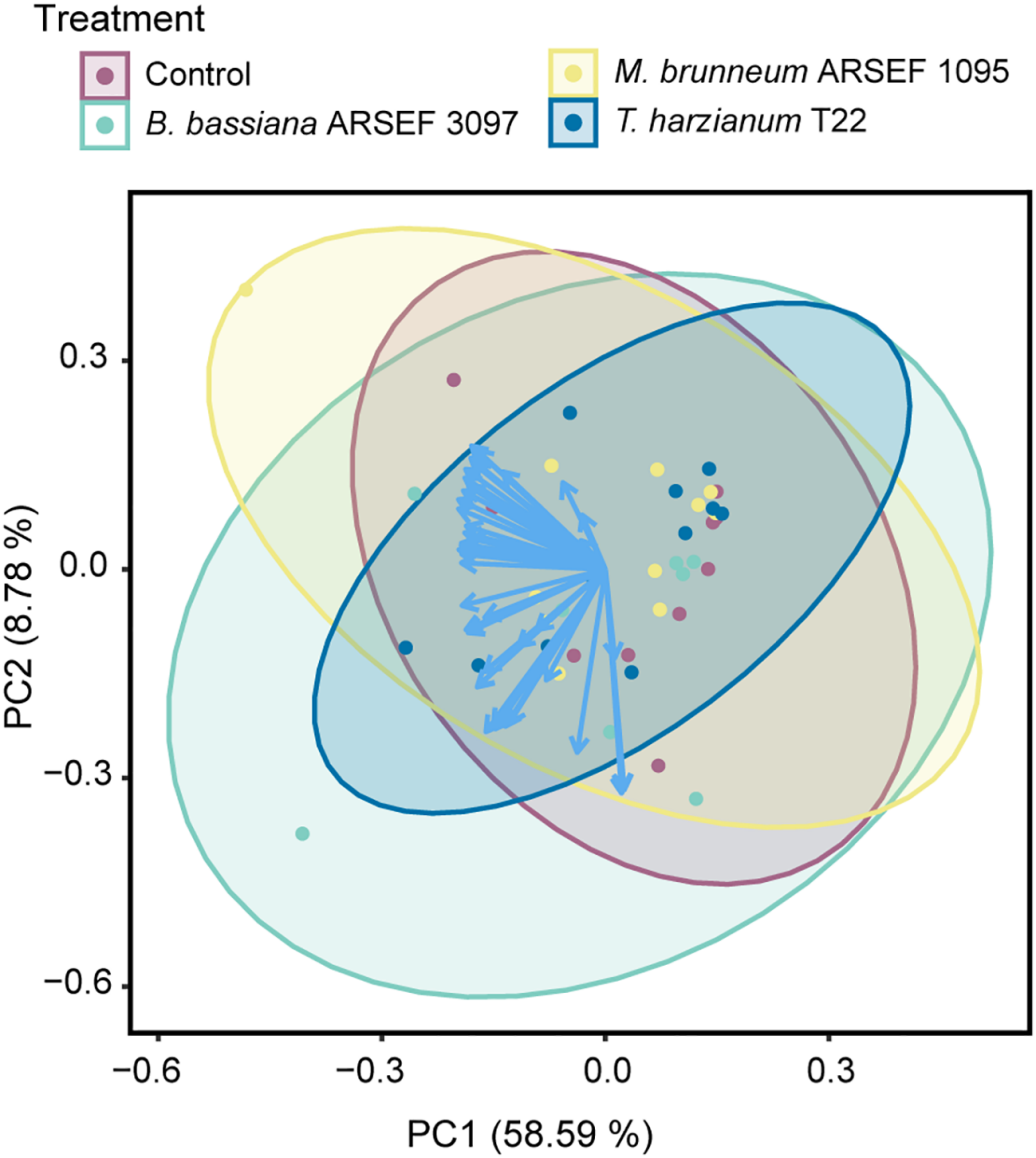
Principal component analysis (PCA) of the volatile composition of tomato plants (cv. Moneymaker) inoculated with *Beauveria bassiana* ARSEF 3097 (green), *Metarhizium brunneum* ARSEF 1095 (yellow), *Trichoderma harzianum* T22 (blue), or non-inoculated (Control, purple). Each data point represents a VOC headspace sample (*n* = 10, except for *Beauveria bassiana* ARSEF 3097 for which *n* = 9). Vectors (in blue) visualize the loadings for each VOC, whereas ellipses represent 95% confidence intervals.

In total, 43 volatile compounds were detected and quantified in the headspace of the tested plants. These consisted predominantly of monoterpenes and sesquiterpenes (**Table 1**). The principal component analysis (PCA) showed no clear separation in VOC composition between the different treatments (**Figure 1**). The first principal component (PC1) in the PCA accounted for 58.59% of the total variation, the second component (PC2) for 8.78% (**Figure 1**). PerMANOVA confirmed that no statistical differences were found between the different treatments (pseudo-*F* = 0.400, *p* = 0.901). As indicated by the PCA, three samples exhibited some deviation from the remaining replicates, i.e. one control sample, one from a plant inoculated with *B. bassiana* ARSEF 3097 and one from a plant inoculated with *M. brunneum* ARSEF 1095. Therefore, the PerMANOVA was also performed with these samples excluded from the dataset. Once again, no statistical differences were found (pseudo-*F* = 0.572, *p* = 0.753). These samples were not omitted in the remainder of the data analysis. When looking at individual volatile compounds, no significant differences were detected between treatments (**Table 1**). To the contrary, when zooming in on the pairwise differences between the control treatment and each of the three fungal treatments, it becomes clear that plants inoculated with *B. bassiana* ARSEF 3097 emitted significantly larger amounts of the sesquiterpenes β-elemene, δ-elemene, α-caryophyllene and β-caryophyllene compared to control plants. No differences were found between the control plants and the other two fungus-treated plants (**Supplementary Table 1**).

**Table 1:** Peak heights^1^ of volatile organic compounds (VOCs) obtained from the headspace of tomato plants inoculated with the fungi *Beauveria bassiana* ARSEF 3097, *Metarhizium brunneum* ARSEF 1095 or *Trichoderma harzianum* T22, compared to mock-inoculated plants (Control).

Compound name and class	ERI^2^	LRI^3^	*B. bassiana* ARSEF 3097	*M. brunneum* ARSEF 1095	*T. harzianum* T22	Control	*P*-value^4^
			(*n* = 9)	(*n* = 10)	(*n* = 10)	(*n* = 10)	
Hydrocarbons							
3,3,5-Trimethylcyclohexene	831	824	692.5 ± 262.2	747.1 ± 334.3	519.2 ± 148.3	443.1 ± 146.6	0.717
3,5,5-Trimethylcyclohexene	841	832	585 ± 220.4	644.8 ± 286.5	433.6 ± 128.4	376.9 ± 126.7	0.725
Monoterpenes							
α-Thujene	932	930	487 ± 115.8	372.5 ± 97.3	489.7 ± 109.7	449 ± 81.6	0.800
α-Pinene	943	939	25936.3 ± 11386	14149.3 ± 1844.6	25434.2 ± 12833.6	15058.1 ± 3322.5	0.928
3,7,7-Trimethylcyclohepta-1,3,5-triene	982	980	104867.3 ± 38427.6	58346.9 ± 23464.9	111092.9 ± 42409.9	60979.4 ± 14865.3	0.605*
β-Pinene	991	989	10518 ± 2708.7	6732.7 ± 1913.3	7747.2 ± 1884.2	7549.5 ± 2023.4	0.639
2-Carene	1008	1007	180899.3 ± 51779.1	139256.3 ± 47161.9	173106.2 ± 43945.5	165207.9 ± 39541	0.883
*m*-Mentha-1,8-diene	1013	1009	381.2 ± 114.8	339.8 ± 122.5	489.6 ± 218.7	339 ± 76.5	0.844
α -Phellandrene	1016	1017	29066.1 ± 12346.8	24454.1 ± 12459.9	27106.4 ± 12135.4	25184 ± 8246.8	0.900*
3-Carene	1019	1019	2725 ± 630.2	2400.1 ± 583	3085.8 ± 861	2432.8 ± 552.2	0.885*
α -Terpinene	1026	1025	23759.1 ± 10263.9	17930.6 ± 9212.9	28798.4 ± 16312.8	22999.1 ± 8332.3	0.859*
*p*-Cymene	1033	1025	21736.2 ± 8334.7	16061.8 ± 7073.8	23784.9 ± 8848.6	18378.1 ± 4382.6	0.745*
(*Z*)-β-Ocimene	1036	1037	911.6 ± 248.1	890.8 ± 233.8	940.8 ± 211.1	963.5 ± 296.3	0.455
Limonene	1039	1038	83322.7 ± 28284.8	58616.8 ± 22299.9	73339.5 ± 21393.5	71661.7 ± 19481.3	0.805
β-Phellandrene	1043	1044	41447.6 ± 12306.4	31963.4 ± 10567.7	39734 ± 9468.3	36913.2 ± 9316.5	0.843
1,8-Cineole	1045	1044	321.6 ± 69.6	235.6 ± 60.9	294.9 ± 62	294.5 ± 54.7	0.599
(*E*)-β-Ocimene	1048	1048	3471.1 ± 828.8	4175 ± 777.4	3903.1 ± 704.9	3160.9 ± 771.6	0.640
γ-Terpinene	1066	1065	4043.4 ± 1406.8	2724.2 ± 1003.8	4226.1 ± 1661.4	3309.7 ± 879.5	0.820*
*m*-Cymene	1089	1085	213.4 ± 52.1	154.8 ± 54.9	352.8 ± 118.7	177.8 ± 32.4	0.679
Isoterpinolene	1095	1091	2117.4 ± 803.8	1283.3 ± 466.5	2163.2 ± 887	1717.3 ± 520.7	0.805*
*p*-Cymenene	1100	1100	2195.5 ± 865.6	2072.6 ± 716.4	3445.7 ± 1269.6	2428.4 ± 578.4	0.635
2,2-Dimethylocta-3,4-dienal	1112	1116	28.7 ± 3.7	344 ± 204	58.2 ± 13.8	38.1 ± 5.9	0.197
*p*-Mentha-1,3,8-triene	1126	1119	356.3 ± 102.3	237.4 ± 40.1	262.7 ± 44.5	330.3 ± 55.9	0.766
Terpinolene	1130	1119	889.4 ± 324.7	751 ± 301.6	1037.7 ± 488.7	649.5 ± 198.5	0.905
2,2,5-Trimethyl-4-cyclohepten-1-one	1138	1149	30.1 ± 5	41.9 ± 7.7	48.3 ± 13.8	45.5 ± 7.3	0.732
*p*-Mentha-1,5,8-triene	1153	1139	628.4 ± 204	402.7 ± 89.3	540.8 ± 97.6	647.2 ± 155.1	0.415
Myrtenol	1194	1194	457.9 ± 149.9	186.5 ± 57.3	399.5 ± 116	509.3 ± 143.7	0.298
Cumin aldehyde	1264	1265	457.2 ± 119.1	424.2 ± 54.6	540.2 ± 180.6	478.1 ± 87.8	0.763
Piperitone	1275	1275	191.2 ± 58.1	152.9 ± 52.2	291.7 ± 124.8	190.1 ± 55.3	0.654
(*Z*)-Myrtanol	1304	1261	152.2 ± 48	116.3 ± 21.5	192.4 ± 86.2	140.3 ± 36.8	0.618
Sesquiterpenes							
δ-Elemene	1355	1357	1713.3 ± 678.1	724.5 ± 338.2	759 ± 264.2	1088.4 ± 365.7	0.124
Isodauca-6,9-diene	1401	1393	1040.8 ± 743.4	1004.9 ± 562.7	363.7 ± 107.9	305.5 ± 72.4	0.815
β-Elemene	1411	1416	192.4 ± 58.1	157.1 ± 51.4	120.6 ± 40.3	150.7 ± 43.2	0.067
β-Caryophyllene	1455	1455	26240.4 ± 16501.7	5310.3 ± 2097	5965.5 ± 2077.7	9338.9 ± 5546.3	0.115*
Guaia-6,9-diene	1469	1450	468.1 ± 169.3	321.5 ± 95.7	292 ± 84.2	333.9 ± 121.6	0.293
α-Caryophyllene	1491	1491	9332.3 ± 5168.7	2213.1 ± 801.4	2754.6 ± 987.4	3118.6 ± 1541.3	0.107*
(*Z*)-β-Guaiene	1515	1513	162.3 ± 56.5	104.4 ± 26.6	111.5 ± 33.9	114.2 ± 32.2	0.251
α-Selinene	1533	1529	163.1 ± 39.9	105.6 ± 19	129.7 ± 32.2	114.3 ± 29.2	0.184
Nitrogen-containing compounds							
2-Methylbutanenitrile	729	729	441 ± 75.3	390.7 ± 110.1	438.4 ± 68.9	547 ± 104.4	0.715
2-Isopropyl-3-methoxypyrazine	1091	1090	166.7 ± 61.1	262.5 ± 107.3	82.9 ± 20.8	216.7 ± 64.5	0.809
Alcohols							
3,3,5-Trimethylcyclohexanol	1064	1073	541.8 ± 262.8	327.1 ± 111.1	225.6 ± 47.7	272.6 ± 78.1	0.591
Ethers							
Anetofuran	1202	1197	1388.1 ± 613.1	864 ± 287.8	1462.3 ± 680.9	1131.8 ± 414.6	0.873*
Homoterpenes							
(*E,E*)-TMTT^5^	1582	1581	390 ± 97.5	268.6 ± 41.1	501.5 ± 162	693 ± 183.4	0.646

^1^Volatile emissions are presented as average peak heights ± SE, divided by 10^3^. The number of replicates is given in parentheses.

^2^ERI = experimentally obtained retention indices.

^3^LRI = retention indices obtained from literature.

^4^P-values are from ANOVA (*) (df = 3, α = 0.05) or Kruskal Wallis.

^5^TMTT = 4,8,12-Trimethyl-1,3,7,11-tridecatetraene.

### 3.2 Effect on insect choice behavior

**Figure 2:**
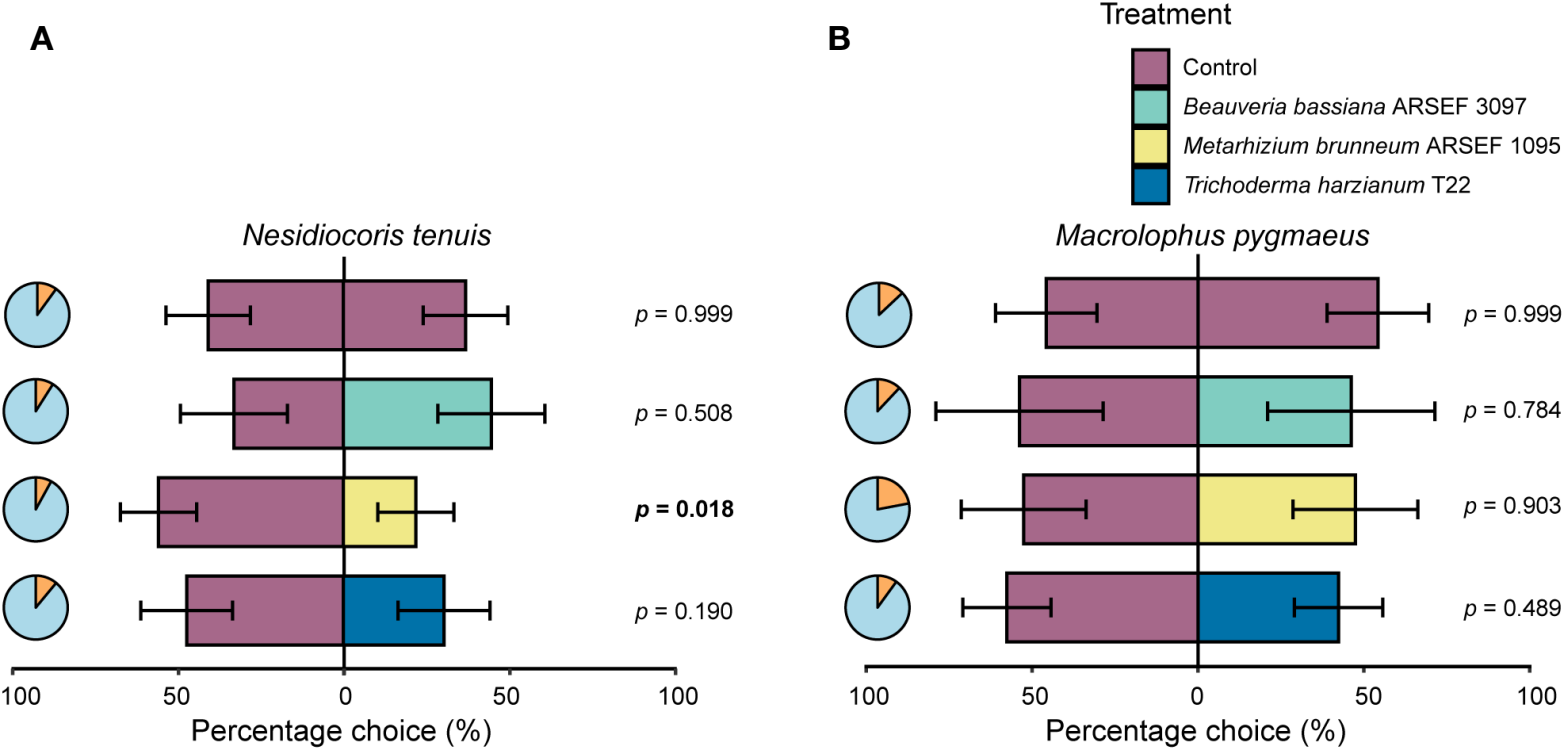
Response (% ± SE) of *Nesidiocoris tenuis* **(A)** and *Macrolophus pygmaeus* **(B)** adult females (tested in ten cohorts of ten females, except for *M. pygmaeus* on *Beauveria bassiana* ARSEF 3097 inoculated plants for which nine cohorts of ten females were included) when given the choice between a non-inoculated tomato plant (cv. Moneymaker) (control, purple) and a fungus-inoculated plant (*Beauveria bassiana* ARSEF 3097 (green), *Metarhizium brunneum* ARSEF 1095 (yellow), or *Trichoderma harzianum* T22 (blue)) in a greenhouse cage assay. *P*-values in bold indicate significant differences in insect response (*p* ≤ 0.05) when compared to a 50:50 distribution. Pie charts show the percentage of responding (blue) and non-responding (orange) insects. Overall responsiveness of *N. tenuis* and *M. pygmaeus* was 90.5% and 85.5%, respectively.

Insect responsiveness in our two-choice bioassay varied between 78 and 92% per tested combinations (**Figure 2**). No significant difference in choice was observed when insects were given a choice between two control plants (*N. tenuis*: percentage choice left plant = 52.5 ± 16.4% versus right plant 47.5 ± 16.4%; *M. pygmaeus*: left plant = 45.7 ± 15.3% versus right plant 54.3 ± 15.3%), demonstrating the robustness of our assay (**Figure 2**). The choice of *N. tenuis* for control versus fungus-inoculated plants varied significantly between fungal treatments (χ²(3) = 16.425, *p* < 0.001). Female *N. tenuis* bugs significantly preferred control plants over *M. brunneum* ARSEF 1095 inoculated plants (*p* = 0.018, percentage choice control plant = 71.8 ± 14.8%) (**Figure 2A**). For the other combinations, no significant differences in choice behavior were recorded. Responses of *M. pygmaeus* did not result in significant differences in choice behavior between control plants and fungus-inoculated plants (χ²(3) = 2.111, *p* = 0.550) (**Figure 2B**).

## 4 Discussion

In this study, we investigated the effect of three fungal strains (*Beauveria bassiana* ARSEF 3097, *Metarhizium brunneum* ARSEF 1095 and *Trichoderma harzianum* T22) on plant volatile emissions and the choice behavior of the generalist zoophytophagous mirids *N. tenuis* and *M. pygmaeus*, with the aim to identify strains that can deter *N. tenuis* while having no effect on the commonly used biocontrol agent *M. pygmaeus*. Selection by insects of suitable feeding sites, mating sites and oviposition sites depends on a unique and complex mixture of plant anatomical and chemical characteristics (Smith and Chuang, 2014). In the early stages of host-seeking and choice behavior, plant VOCs play a key role in guiding insects to suitable food plants (von Arx et al., 2012). Plants associate with diverse microorganisms that form intimate relationships with their hosts (Tian et al., 2020). As some of them may affect host physiology and functioning (Vidal and Jaber, 2015; Vega, 2018; Gange et al., 2019), we hypothesized that inoculation with plant-beneficial fungi affects VOC composition and host-plant selection.

Our results show that the fungi tested did not significantly alter the VOC composition of tomato plants. By contrast, Wilberts et al. (2022) found that inoculation with the entomopathogenic fungus *Akanthomyces muscarius* Petch ARSEF 5128 (Hypocreales: Cordycipitaceae) significantly changed the VOC composition of sweet pepper compared to non-inoculated plants. Inoculation with *B. bassiana* ARSEF 3097, i.e. the strain also used in this study, however, did not change the VOC profile of sweet pepper (Wilberts et al., 2022). Together with our study, this suggests that *B. bassiana* ARSEF 3097 has no or only a limited impact on plant odor. When sweet pepper plants were inoculated with *A. muscarius*, significantly higher amounts of β-pinene were emitted than non-inoculated plants, and significantly higher amounts of indole than *B. bassiana*-inoculated and non-inoculated plants. Notably, the authors found that *A. muscarius* inoculated plants were more attractive to aphids than control plants, most probably because of the altered VOC composition (Wilberts et al., 2022). In line with our findings, *T. harzianum* T22 was not found to alter the VOC composition of tomato plants as long as they were not infested by aphids (Coppola et al., 2017). When plants were infested with aphids, the VOC composition of fungus-inoculated plants was different from infested control plants, which coincided with an increased attraction of the aphid parasitoid *Aphidius ervi* Haliday (Hymenoptera: Braconidae). This was linked to an upregulation of genes involved in terpenoid biosynthesis and the salicylic acid-mediated defense pathway, which led to increased volatile emission levels of methyl salicylate and β-caryophyllene (Coppola et al., 2017).

Whereas no differences in VOC blends were found between the different fungi, the results of our bioassays showed that when *N. tenuis* females were given the choice between control plants and fungus-inoculated plants, *N. tenuis* preferred control plants over *M. brunneum* ARSEF 1095 inoculated plants. Among the tested *N. tenuis* individuals, 72% chose the control treatment. In all other combinations tested, no significant differences were found, neither for *N. tenuis* nor for *M. pygmaeus*. This is a promising result for future biocontrol strategies, where *N. tenuis* may be deterred from the crop when inoculated with *M. brunneum* ARSEF 1095 and attracted to non-inoculated plants where they can be concentrated and locally treated with a pesticide, while *M. pygmaeus* remains undisturbed. However, it has to be noted that insect recordings were made at only one time interval, i.e. 24 h after insect release, and that no data are available for other time points. The mechanisms behind the observed aversion of *N. tenuis* for *M. brunneum* inoculated plants are still unclear. No differences were found in the emissions of individual compounds between the control plants and plants inoculated with *M. brunneum* ARSEF 1095, suggesting that causal compounds were below the detection limit or that other factors are involved. Previous research has shown that host-selection by herbivores is not only driven by olfactory cues, but also by visual, contact and gustatory cues (Bernays and Chapman, 2007). Additional research is required to find out whether and how these cues are influenced by fungal inoculation. Nevertheless, it has to be noted that insect behavior is not always influenced by the volatile blend as a whole or the presence and abundance of specific compounds in the blend, but often depends on the level and ratio of the different compounds (Bruce and Pickett, 2011; Uefune et al., 2013; Goelen et al., 2021).

In our study insects were allowed to see and probe the plants before settling. In order to purely evaluate olfactory responses, olfactometer bioassays could be performed in which visual cues and other potential stimuli are eliminated (Naselli et al., 2017; Wilberts et al., 2022). In previous research, it was shown that inoculation of Moneymaker tomato plants with *M. brunneum* ARSEF 1095 significantly changed the nutritional profile of the tomato plants. For example, *M. brunneum* ARSEF 1095 significantly reduced the total content of micronutrients and total nitrogen content in the sap of tomato leaves compared to control plants (Meesters et al., 2023). Potentially, this may have contributed to the deterrent effect on *N. tenuis* adults over the investigated period of 24 hours. Furthermore, the production of secondary metabolites, produced by or induced by the fungus, might be another reason for the difference in the choice behavior (Pieterse et al., 2014; Quesada Moraga, 2020). In potato *Solanum tuberosum* Linnaeus (Solanales, Solanaceae) plants, for example, it was shown that *M. brunneum* ARSEF 1095 produced secondary metabolites such as destruxin A during transient endophytic colonization (Ríos-Moreno et al., 2016), which has insecticidal activities and may suppress the insect’s innate immune system (Hu et al., 2007; Pal et al., 2007). Further research is needed to find out whether secondary metabolites may help explain the change in *N. tenuis* behavior following fungal inoculation (Shrivastava et al., 2015).

Among the VOCs detected in this study, monoterpenes and sesquiterpenes were the most representative classes. By contrast, no green leaf volatiles (GLVs) were detected in this study. GLVs are six carbon (C6) compounds that are typically released in response to mechanical damage, herbivore feeding or pathogen attack, but also as a consequence of abiotic stresses (Ameye et al., 2018). The fact that no GLVs were found suggests that they were not induced after inoculation with beneficial fungi, confirming previous studies (Papantoniou et al., 2022). However, there are also studies showing that plant beneficial fungi can enhance the production of GLVs, as for example shown for arbuscular mycorrhizal fungi (Velásquez et al., 2020). From all sesquiterpenes detected in our study, β-caryophyllene represented the highest level in *B. bassiana* ARSEF 3097 inoculated plants compared to control plants (**Table 1**). β-Caryophyllene has been reported to have negative growth regulatory effects, as well as contact and fumigant toxicity against agricultural pests (Ma et al., 2020; Mahajan et al., 2022). In addition, plants overexpressing the β-caryophyllene synthase gene have been reported to reduce pest populations such as cotton aphid *Aphis gossypii* Linnaeus (Hemiptera: Ahididae), cotton bollworm *Helicoverpa armigera* Hübner (Lepidoptera: Noctuidae), the herbivorous mirid *Apolygus lucorum* Meyer-Dür (Hemiptera: Miridae) and the common cutworm *Spodoptera litura* Fabricius (Lepidoptera: Noctuidae), while parasitoids such as *Peristenus spretus* Chen & van Achterberg (Hymenoptera: Braconidae) and *Aphidius gifuensis* Ashmead (Hymenoptera: Braconidae) are attracted (Zhang et al., 2020; Mahajan et al., 2022). Although no significant effect of plant inoculation with *B. bassiana* ARSEF 3097 on host-selection behavior was observed, and generalizations over different cultivars must be made with caution, inoculation of tomato cultivar Micro-Tom with *B. bassiana* ARSEF 3097 in previous experiments was found to reduce *N. tenuis* feeding damage and increase its mortality rate (Meesters et al., 2023). Undoubtedly, further investigation is required, yet the increased level of β-caryophyllene might potentially have played a role here.

To conclude, our study has demonstrated that the three tested plant-beneficial fungi have no substantial impact on the VOC profiles in tomato (cv. Moneymaker). Nevertheless, altered host selection behavior was observed for *N. tenuis* when plants were inoculated with *M. brunneum* ARSEF 1095. Specifically, *N. tenuis* was deterred by fungus-inoculated plants, while no effects were observed for the closely related biocontrol agent *M. pygmaeus*. Further research is required to investigate whether these findings may lead to new biocontrol strategies against *N. tenuis*.

## Data availability statement

The original contributions presented in the study are included in the article/**Supplementary Material**. Further inquiries can be directed to the corresponding author.

## Ethics statement

The manuscript presents research on animals that do not require ethical approval for their study.

## Author contributions

CM: Conceptualization, Data curation, Formal analysis, Investigation, Methodology, Project administration, Visualization, Writing – original draft, Writing – review & editing. BW: Formal Analysis, Validation, Writing – review & editing. MD: Resources, Validation, Writing – review & editing. HJ: Conceptualization, Funding acquisition, Project administration, Supervision, Validation, Writing – review & editing. BL: Conceptualization, Funding acquisition, Project administration, Supervision, Validation, Writing – review & editing.
