# Supplementary material for: Limited effects of plant-beneficial fungi on plant volatile composition and host-choice behavior of Nesidiocoris tenuis
Source: Front Plant Sci. 2024 Jan 3;14:1322719. doi: 10.3389/fpls.2023.1322719 (PMC10791865; doi:10.3389/fpls.2023.1322719)
Supplement: Supplementary file 1 [file DataSheet_1.pdf]

Supplementary Material

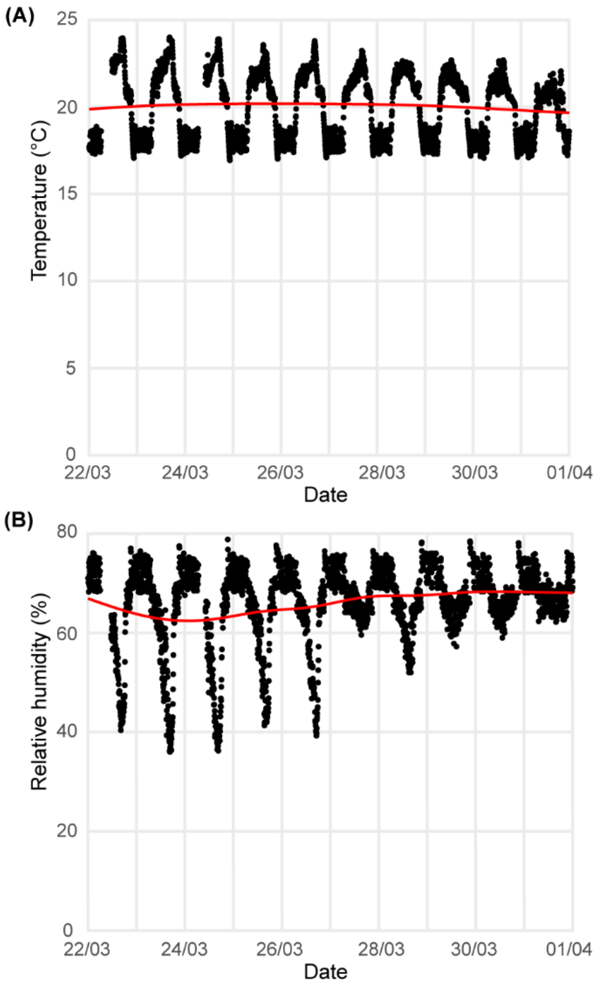

Supplementary Figure 1 Recorded temperature **(A)** and relative humidity **(B)** inside the greenhouse compartment during the experiments performed in this study (two-choice assay). Each data point represents a measurement every five minutes, while the red line represents the daily mean.

Supplementary Table 1. *P*-values[1] of univariate significance tests for all volatile organic compounds (VOCs) detected in this study. *P*-values ($p \leq 0.05$, in bold) indicate that the emission of that compound differs significantly between the two compared treatments. Plants were inoculated with *Beauveria bassiana* ARSEF 3097, *Metarhizium brunneum* ARSEF 1095 or *Trichoderma harzianum* T22 or mock-inoculated with physiological saline solution (Control).

Compound name and class	*B. bassiana* ARSEF 3097 vs Control	*M. brunneum* ARSEF 1095 vs Control	*T. harzianum* T22 vs Control
Hydrocarbons			
3,3,5-Trimethylcyclohexene	0.780	0.842	0.605
3,5,5-Trimethylcyclohexene	0.842	0.905	0.557
Monoterpenes			
α-Thujene	0.400	0.447	0.785*
α-Pinene	0.661	0.720	0.973
3,7,7-Trimethylcyclohepta-1,3,5-triene	0.400	0.315	0.705
β-Pinene	0.278	0.604	0.918
2-Carene	0.604	0.447	0.557
m-Mentha-1,8-diene	0.604	0.356	0.557
α-Phellandrene	0.720	0.400	0.654
3-Carene	0.604	0.563*	0.986*
α-Terpinene	0.780	0.447	0.468
p-Cymene	0.604	0.211	0.349
(*Z*)-β-Ocimene	0.356	0.838	0.572
Limonene	0.400	0.356	0.605
β-Phellandrene	0.549	0.356	0.557
1,8-Cineole	0.156	0.211	0.705
(*E*)-β-Ocimene	0.624*	0.902	0.833
γ-Terpinene	0.356	0.447	0.654
m-Cymene	0.683	0.270	0.418

Isoterpinolene	0.549	0.497	0.512
p-Cymenene	0.438	0.205	0.307
2,2-Dimethylocta-3,4-dienal	0.141	0.720	0.084
p-Mentha-1,3,8-triene	0.713	0.235	0.525
Terpinolene	0.661	0.549	0.605
2,2,5-Trimethyl-4-cyclohepten-1-one	0.204*	0.624	0.438
p-Mentha-1,5,8-triene	0.327	0.182	0.192
Myrtenol	0.182	0.549	0.085
Cumin aldehyde	0.400	0.391	0.672
Piperitone	0.780	0.278	0.314
(*Z*)-Myrtanol	0.720	0.549	0.485*
Sesquiterpenes			
δ-Elemene	**0.028**	1.000	0.573
Isodauca-6,9-diene	0.413	0.713	1.000
β-Elemene	**0.037**	0.744	0.672
β-Caryophyllene	**0.035**	0.661	0.973
Guaia-6,9-diene	0.095	0.567	0.481
α-Caryophyllene	**0.043**	0.905	0.973
(*Z*)-β-Guaiene	0.079	0.744	0.481
α-Selinene	0.079	1.000	0.972
Nitrogen-containing compounds			
2-Methylbutanenitrile	0.497	0.278	0.426
2-Isopropyl-3-methoxypyrazine	1.000	0.653	0.805
Alcohols			
3,3,5-Trimethylcyclohexanol	0.356	0.683	0.809
Ethers			
Anetofuran	0.549	0.780	0.918
Homoterpenes			
(*E*,*E*)-TMTT[2]	0.245*	0.653	0.251

[1]*P*-values of Student's *t*-test (*) or Wilcoxon Rank Sum test, for compounds meeting assumptions for normality or non-normality, respectively. Compounds with significant differences (p-value < 0.05) between treatments are indicated in bold.
[2] TMTT = 4,8,12-Trimethyl-1,3,7,11-tridecatetraene.
